# Flavonoids and anthocyanins in seagrasses: implications for climate change adaptation and resilience

**DOI:** 10.3389/fpls.2024.1520474

**Published:** 2025-01-28

**Authors:** Jana Botes, Xiao Ma, Jiyang Chang, Yves Van de Peer, Dave Kenneth Berger

**Affiliations:** ^1^ Department of Plant and Soil Sciences, University of Pretoria, Pretoria, South Africa; ^2^ Forestry and Agricultural Biotechnology Institute, University of Pretoria, Pretoria, South Africa; ^3^ Department of Plant Biotechnology and Bioinformatics, Ghent University, Ghent, Belgium; ^4^ Centre for Plant Systems Biology, VIB, Ghent, Belgium; ^5^ Department of Biochemistry, Genetics and Microbiology, University of Pretoria, Pretoria, South Africa; ^6^ College of Horticulture, Academy for Advanced Interdisciplinary Studies, Nanjing Agricultural University, Nanjing, China

**Keywords:** seagrasses, ocean warming, flavonols, chalcone synthase, phenylpropanoids, phenols, chemical ecology, marine angiosperms

## Abstract

Seagrasses are a paraphyletic group of marine angiosperms and retain certain adaptations from the ancestors of all embryophytes in the transition to terrestrial environments. Among these adaptations is the production of flavonoids, versatile phenylpropanoid secondary metabolites that participate in a variety of stress responses. Certain features, such as catalytic promiscuity and metabolon interactions, allow flavonoid metabolism to expand to produce novel compounds and respond to a variety of stimuli. As marine environments expose seagrasses to a unique set of stresses, these plants display interesting flavonoid profiles, the functions of which are often not completely clear. Flavonoids will likely prove to be effective and versatile agents in combating the new host of stress conditions introduced to marine environments by anthropogenic climate change, which affects marine environments differently from terrestrial ones. These new stresses include increased sulfate levels, changes in salt concentration, changes in herbivore distributions, and ocean acidification, which all involve flavonoids as stress response mechanisms, though the role of flavonoids in combatting these climate change stresses is seldom discussed directly in the literature. Flavonoids can also be used to assess the health of seagrass meadows through an interplay between flavonoid and simple phenolic levels, which may prove to be useful in monitoring the response of seagrasses to climate change. Studies focusing on the genetics of flavonoid metabolism are limited for this group, but the large chalcone synthase gene families in some species may provide an interesting topic of research. Anthocyanins are typically studied separately from other flavonoids. The phenomenon of reddening in certain seagrass species typically focuses on the importance of anthocyanins as a UV-screening mechanism, while the role of anthocyanins in cold stress is discussed less often. Both of these stress response functions would be useful for adaptation to climate change-induced deviations in tidal patterns and emersion. However, ocean warming will likely lead to a decrease in anthocyanin content, which may impact the performance of intertidal seagrasses. This review highlights the importance of flavonoids in angiosperm stress response and adaptation, examines research on flavonoids in seagrasses, and hypothesizes on the importance of flavonoids in these organisms under climate change.

## 1 Introduction

Seagrasses are a paraphyletic group of marine angiosperms consisting of approximately 72 species (Unsworth et al., 2022), all belonging to the order Alismatales, which includes 11 freshwater and 4 fully marine families. The four seagrass families (the Posidoniaceae, Zosteraceae, Hydrocharitaceae, and Cymodoceaceae) (Effrosynidis et al., 2019; Pfeifer and Classen, 2020) arose from at least three independent lineages transitioning from freshwater to a marine habitat, which has not occurred in any other angiosperm lineage (Wissler et al., 2011; Chen et al., 2022). Despite the limited species diversity, seagrasses boast a large distribution, spanning an area of over 160,000 km^2^ along most of the world’s temperate and tropical coastlines in large meadows (McKenzie et al., 2020; Short et al., 2007). Seagrasses are of great importance to marine ecosystems and the planet, providing a variety of ecosystem services such as carbon sequestration (Duarte and Krause-Jensen, 2017; Serrano et al., 2021; Marbà et al., 2015), sediment production (East et al., 2023; Keulen and Borowitzka, 2003), food and habitat for vertebrate and invertebrate marine life (Jackson et al., 2015; Gartner et al., 2013; Bloomfield and Gillanders, 2005), and many others, as reviewed by Mtwana Nordlund et al. (2016). Besides human coastal development and activities causing loss of seagrasses (Short et al., 2006), climate change also threatens seagrasses and their ecosystem services, as it is predicted to have a profound impact on seagrass endemism and distribution, with many species becoming more restricted and meadows shifting to currently unprotected areas (Daru and Rock, 2023; Marbà et al., 2015). Understanding the stress response systems of seagrasses will aid us in predicting the extent to which seagrasses will tolerate the coming climatic changes.

The ocean has absorbed the majority of the heat gained over the last 50 years of global warming, mostly in the upper 700 m, leading to ocean warming, with an average increase of 0.9°C in this upper layer during the 20th century (Domingues et al., 2008). Other impacts of anthropogenic climate change on the ocean include ocean acidification due to increased CO_2_ levels, increased stratification, intensified storms, rising sea levels, and hypoxia (Hoegh-Guldberg and Bruno, 2010; Venegas et al., 2023). Although increased CO_2_ levels should benefit seagrasses as photosynthetic organisms (Zimmerman et al., 2017; Listiawati and Kurihara, 2021), it is outweighed by the negative impact of high temperatures from ocean warming and marine heatwaves; ocean warming is therefore expected to be the most important factor impacting seagrass health and distribution (Diaz-Almela et al., 2007; Repolho et al., 2017; Moore et al., 2012; Zhang et al., 2022; Short et al., 2016). It is predicted that climate change will cause massive shifts in the distributions of different seagrass species due to shifts in climatically suitable environments, which will negatively impact species that rely on seagrass meadows as a food source or habitat (Daru and Rock, 2023; Duarte et al., 2018). Seagrasses, unlike seaweed which occupies a similar ecological niche, are angiosperms and therefore descended from land plants. This unique ancestry affords them certain traits that may aid them in resisting some of the stress imposed by climate change.

The transition from an aquatic to a terrestrial habitat by the ancestors of all land plants required several key adaptations, as functions previously fulfilled by the surrounding aqueous medium, such as UV screening and mechanical support, now required adaptive mechanisms from the organism, leading to the advent of compounds such as flavonoids and lignins (De Vries and Archibald, 2018). Seagrasses, descended from terrestrial angiosperms, retain certain adaptations from their ancestors’ switch to terrestrial ecosystems, which have greatly expanded in the angiosperms in the millennia on land. Seagrasses therefore possess traits for a variable terrestrial environment in a more stable marine environment, which presents a completely different host of stresses, such as high levels of salinity, a shifting substrate, and low light (Ma et al., 2024). While some adaptations to terrestrial life are lost in seagrasses, like stomata which are not beneficial outside of a gaseous environment (Papenbrock, 2012; Chater et al., 2017; Larkum et al., 2017), other adaptations are retained and may actually be beneficial in a marine environment. Among these retained adaptations are the flavonoids, a group of secondary metabolites restricted to the embryophytes, which function in a variety of stress responses and are hypothesized to have been important in the shift to terrestrial ecosystems by the ancestors of land plants. With these compounds and the genes underlying their biosynthesis, seagrasses maintain a powerful and flexible repertoire of stress-response compounds, which are very useful to plants inhabiting high-stress environments and may play an important role in allowing them to survive the conditions imposed by climate change (Di Ferdinando et al., 2014; Laoué et al., 2022). This review discusses the evolution and function of flavonoids and how they might be beneficial to seagrasses as angiosperms in a marine environment under conditions of climate change.

## 2 Origin of flavonoids

**Figure 1:**
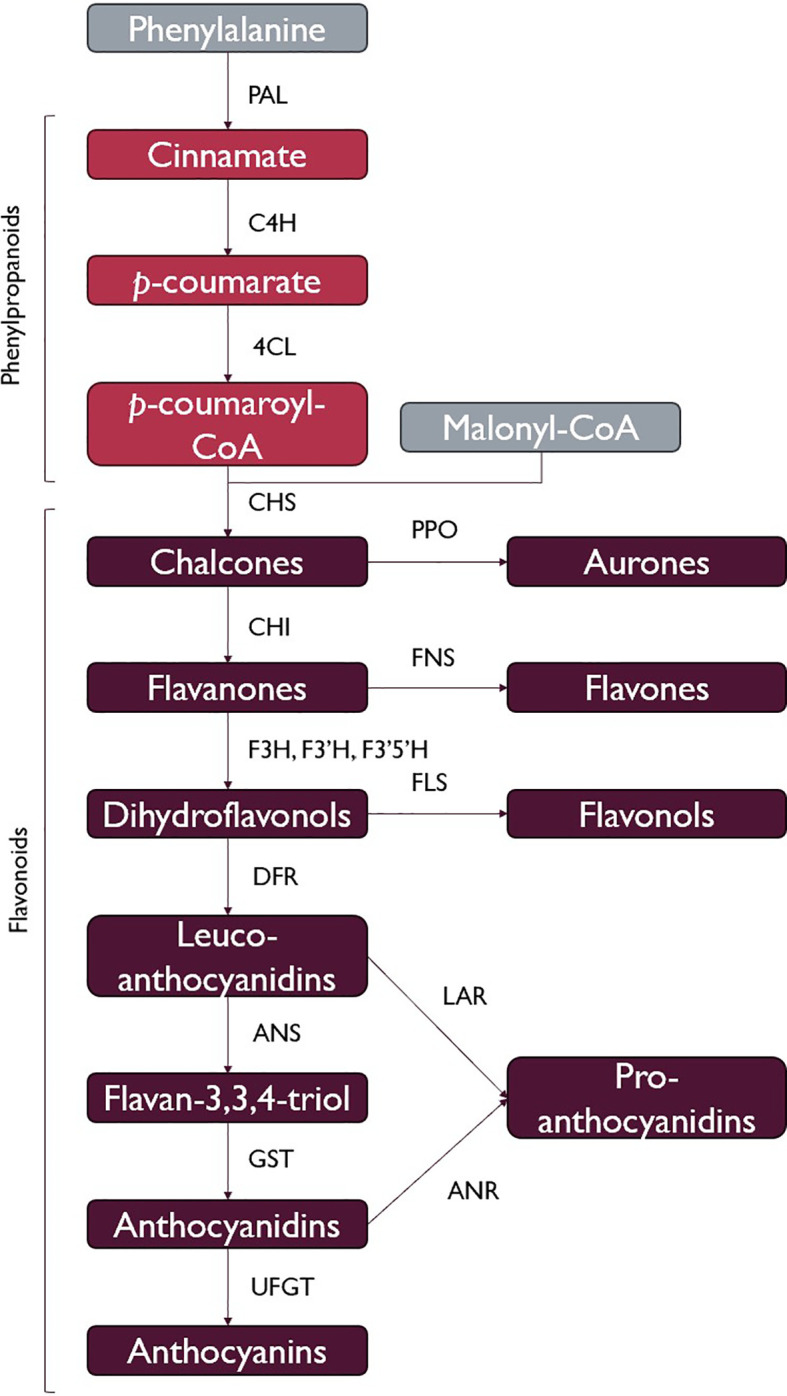
A simplified view of flavonoid biosynthesis. Flavonoids are derived from the phenylpropanoid pathway, as p-coumaroyl-CoA is a product of the phenylpropanoid pathway (phenylalanine ammonia-lyase (PAL), cinnamic acid 4-hydrolase (C4H), p-coumaroyl:coA ligase (4CL)). Chalcone synthase (CHS) catalyses the first committed step of flavonoid biosynthesis, and regulation of CHS therefore regulates flavonoid production. Other enzymes of the pathway include polyphenol oxidase (PPO), chalcone isomerase (CHI), flavone synthase (FNS), flavanone 3-hydroxylase (F3H), flavonoid 3’- and flavonoid 3’5’-hydroxylase (F3’H and F3’5’H), flavonol synthase (FLS), dihydroflavonol reductase (DFR), anthocyanidin synthase (ANS), anthocyanin-related glutathione S-transferase (GST), leucoanthocyanidin reductase (LAR), anthocyanidin reductase (ANR), UDP-glucose:flavonoid 3-O-glucosyltransferase (UFGT). Figure simplified from Eichenberger et al. (2023), following the discovery of the catalytic involvement of GST in anthocyanin biosynthesis. This is not a comprehensive list of all flavonoids, and many types of flavonoids are only produced in a handful of lineages.

The phenylpropanoid pathway allows for the production of flavonoids and lignin, which are both argued to be vital elements in allowing the ancestor of land plants to survive in terrestrial environments (Weng and Chapple, 2010; Kenrick and Crane, 1997; De Vries and Archibald, 2018). In this pathway, phenylalanine is converted to trans-cinnamic acid through the action of phenylalanine ammonia-lyase (PAL), which is then converted to p-coumaric acid by cinnamic acid 4-hydrolase (C4H). p-coumaroyl:CoA ligase (4CL) can then produce p-coumaroyl-CoA from p-coumaric acid, which can be used for lignin and flavonoid biosynthesis. This pathway is ubiquitous among land plants, which supports the importance of flavonoids and lignin in the move to terrestrial environments. PAL seems to have been acquired by horizontal gene transfer during symbiotic interactions with soil bacteria or fungi and most likely functioned in the production of antimicrobial or UV-protectant secondary metabolites (Emiliani et al., 2009). Three malonyl-CoA molecules and a p-coumaroyl-CoA produced by 4CL are then used to produce chalcones through the action of chalcone synthase (CHS), a type III polyketide synthase, which marks the beginning of flavonoid biosynthesis (**Figure 1**). There are several classes of flavonoids comprising thousands of different molecules, namely, aurones, flavones, flavonols, proanthocyanidins, and anthocyanins, with these compounds all being synthesized by branches of flavonoid metabolism which share in enzymes to differing degrees, leading to competition for reaction intermediates. Flavonoids are found in vascular plants, mosses, and liverworts, and although flavonoids have been detected in various algae (Goiris et al., 2014), the quantities are extremely low compared to the typical contents of land plants (Davies et al., 2020), and the diversity is very low, being mostly limited to the flavone apigenin (Goiris et al., 2014). Type III PKS genes have been identified in some algal lineages, including homologs for CHS, as well as homologs for 4CL (De Luca and Lauritano, 2020). Some of these taxa, however, are missing homologs for other key genes involved in cinnamic and coumaric acid biosynthesis, which are important intermediates for the synthesis of the CHS substrate (Jiao et al., 2020). It is not unlikely for flavonoids to have first appeared in photosynthetic marine organisms, where they had limited diversity and functionality and served as an excellent exaptation for the transition to terrestrial habitats, where selection favored their increased production and diversification (De Vries and Archibald, 2018).

### 2.1 Flavonoids in terrestrial plants

It is hypothesized that the success of plants in colonizing terrestrial environments is coupled with the advent of flavonoids, due to their ubiquitous distribution and incredible functional diversity (Kenrick and Crane, 1997; Weng and Chapple, 2010). A few classes of flavonoids and their biosynthesis are summarized in **Figure 1**. Flavonoids are involved in biotic and abiotic stress responses (Fini et al., 2011; Dong and Lin, 2021), hormonal regulation (Peer et al., 2004), and signaling for microbial and animal interactions (Rahme et al., 2014; Shimada et al., 2003; Dong and Lin, 2021). There are two classic hypotheses for the initial role performed by flavonoids in early land plants. One hypothesis is that the importance of flavonoids in the colonization of terrestrial environments comes from their UV-B screening properties (Li et al., 1993). UV-B radiation decreases photosynthetic efficiency by causing damage to photosystem II (Szilárd et al., 2007) and leads to the production of reactive oxygen species (ROS), which can damage DNA (Li et al., 2002). This hypothesis was based on flavonoid-deficient mutants, showing an increase in sensitivity to UV-B radiation (Li et al., 1993) and the accumulation of flavonoids in the epidermis in response to UV-B exposure (Tevini et al., 1991).

There are several pieces of evidence that oppose this hypothesized initial function of flavonoids. The efficacy of flavonoids as UV-screening compounds requires their accumulation in high quantities, which would not have been possible without certain enzymes to transport them to the cell wall and vacuole, and these enzymes likely would not have existed when flavonoids first arose (Stafford, 1991). This does not discredit the importance of UV-B defense for survival on land, but non-flavonoid compounds may have fulfilled this role, with the UV-B screening role of flavonoids being an exaptation of these molecules when novel enzymatic functions allowed them to accumulate at high levels. Indeed, hydroxycinnamates, which also belong to the phenylpropanoid class of secondary metabolites, can effectively absorb UV-B radiation and play a role in UV-B screening alongside flavonols (Burchard et al., 2000). Lignins may also have played a role in photoprotection in early land plants: lignin epitopes have been identified in the cell walls of bryophytes and are not associated with vascular or mechanical tissues (Ligrone et al., 2008). Non-flavonoid-producing organisms, including fungi, cyanobacteria, algae, lichens, and dinoflagellates, employ mycosporine-like amino acids to protect photosystem II against UV damage (Taira and Taguchi, 2017) and macromolecules against ROS, thereby playing the same role in this stress response as flavonoids in plants. Their biosynthesis also seems to be derived from the shikimate pathway, further tying them to the phenylpropanoids, which are derived from the chorismate produced by this pathway.

The second hypothesis is that flavonoids first arose to function as signaling molecules, specifically in the regulation of auxins (Stafford, 1991). Stafford argued for this role of flavonoids in light of the arguments outlined above, as flavonoids would have been able to perform signaling functions at low cytoplasmic concentrations, and other compounds existed in the ancestors of land plants that functioned in UV-B screening. Many flavonoid-deficient mutant angiosperms show altered auxin-related development, including dwarfing, altered root development, and loss of pollen viability under temperature stress (Dare and Hellens, 2013; Muhlemann et al., 2018; Maloney et al., 2014), though these traits and their severity vary between flavonoid-deficient mutants (Burbulis et al., 1996). Flavonols, specifically, are important in regulating auxin transport during nodulation to promote organogenesis (Zhang et al., 2009; Ng et al., 2015), and flavonoids are also involved in the microbial signaling required with root nodulation (Abdel-Lateif et al., 2012). The formation of microbial symbiotic interactions and improved nutrient acquisition were important innovations for the success of plants in terrestrial environments, with flavonoids being involved in both, as reviewed by Hassan and Mathesius (2012). The relationship between auxin transport and flavonoids has also been observed in gymnosperms (Ramos et al., 2016), but there is no evidence that flavonoids play similar roles in the developmental processes of bryophytes, with flavonoid-deficient liverwort mutants showing normal development (Clayton et al., 2018). It therefore cannot be assumed that the ancestral role of flavonoids was auxin regulation.

The first hypothesis has since been adopted, placing the emphasis on the involvement of flavonoids in UV-B stress on their antioxidant activity, rather than their screening properties (Fini et al., 2011; Agati et al., 2013). The ROS-scavenging function of flavonoids also explains their production in response to a host of biotic and abiotic stresses, with chalcone synthase, which catalyzes the first committed reaction in the synthesis of all flavonoids, being upregulated in response to heat and cold stress (Shvarts et al., 1997), UV-B radiation and blue light (Christie and Jenkins, 1996), wounding (Richard et al., 2000), and biotic stress (Dao et al., 2011), which all lead to the accumulation of reactive oxygen species. Acting as ROS-scavenging compounds would not require the accumulation of flavonoids in the vacuole or cell wall but would still allow flavonoids to reduce the damage sustained as a result of increased UV-B exposure when the ancestors of land plants moved to a terrestrial environment.

### 2.2 Expansion of flavonoid metabolism

Like many other pathways of secondary metabolism, gene duplication events are important in the expansion of flavonoid metabolism and the origin of new enzymes and branchpoints. Some of the important biosynthetic genes arose from enzymes involved in primary metabolism, such as CHI, which is most likely descended from a non-catalytic fatty acid-binding protein (Ngaki et al., 2012; Kaltenbach et al., 2018). This also allows for neofunctionalization, with stilbene synthases having arisen from CHS multiple times independently, a process that requires only a few missense mutations (Tropf et al., 1994). Legume-specific type II CHI enzymes also originate from the typical type I CHI, which allows for the synthesis of flavonoid compounds required for microbial signaling (Shimada et al., 2003). FNS I likely arose from F3H, allowing for flavone synthesis in the Apiaceae (Martens et al., 2003; Gebhardt et al., 2005).

The enzymes involved in flavonoid biosynthesis, shown in **Figure 1**, are arranged in a metabolon on the cytoplasmic face of the endoplasmic reticulum. The arrangement of enzymes within the metabolon differs between species but is also determined by spatial–temporal expression differences, with some proteins not being present in the metabolon and increased expression of certain enzymes in certain tissues allowing them to outcompete certain other enzymes for shared binding sites (Crosby et al., 2011). The patterns of these interactions are important for the branchpoints of flavonoid metabolism: for example, DFR and FLS compete for dihydroflavonol substrates at the branchpoint between anthocyanin and flavonol synthesis and additionally compete to interact with CHS within the metabolon (Crosby et al., 2011). These competitive interactions are important in determining the flux of intermediates to different branches of the pathway (Watkinson et al., 2018), especially considering the catalytic promiscuity exhibited by many of the enzymes involved in flavonoid metabolism. For some of the enzymes, like F3H, the catalytic promiscuity is more apparent *in vitro*, as protein–protein interactions within the metabolon aid in channeling the appropriate substrates from one enzyme to the next in the pathway. It can therefore be hypothesized that the ability to interact with other proteins of the metabolon is what shapes the pathway, rather than the substrate and product specificity of the individual enzymes. These factors have allowed for the expansion of flavonoid metabolism when plants adapt to new environments and the advent of novel useful flavonoid functions, which is likely why flavonoids are so well conserved in the land plants and could well have benefited the ancestors of seagrasses when returning to a marine environment.

### 2.3 Flavonoids in seagrasses

Seagrasses present an interesting case when discussing the various roles performed by flavonoids, as these aquatic higher plants are descended from terrestrial ancestors, with flavonoid production being an ancestral trait and an adaptation toward life on land. Generally, flavonoids perform similar functions in seagrasses compared to land plants, being involved in oxidative stress responses and UV screening —the versatility of these compounds has allowed them to remain relevant outside of the environment in which they originally arose, with the versatility of the biosynthetic pathway allowing for new compounds to evolve.

There have been several studies characterizing the flavonoid contents of various species of seagrasses, with sulfated flavonoids being of particular prevalence in this group (McMillan et al., 1980). Sulfated flavones have been characterized in the genera *Zostera*, *Thalassia*, *Halophila*, *Enhalus*, and *Phyllospadix*, with some other genera producing sulfated non-flavonoid phenolic acids (McMillan et al., 1980). Sulfation greatly increases the solubility of these compounds, even more so than the corresponding glycone, which may be related to the function of these compounds (Grignon-Dubois and Rezzonico, 2018). Sulfated flavonoids may be important for the storage of inorganic sulfates in a more soluble form, facilitating transport through the plant cell and allowing for extrusion (Harborne, 1977; Grignon-Dubois and Rezzonico, 2023), as the toxic sulfide ions present in high concentrations in seawater has little effect on the health of certain seagrass species (Hasler-Sheetal and Holmer, 2015). *Posidonia oceanica*, for example, is much more sensitive to sulfides than *Zostera marina* or *Thalassia testudinum*, which both produce sulfated flavonoids (Grignon-Dubois and Rezzonico, 2018).

Sulfated flavonoids are not limited to seagrasses and are strongly associated with plants growing near water bodies rich in mineral salts, indicating that these compounds might act as an adaptation for dealing with high salt exposure (Teles et al., 2018; Harborne, 1975). Most plant families that produce these compounds are not closely related, indicating that this trait arose multiple times independently through the evolution of different sulfotransferases, though flavonols and flavones are generally the targets for sulfation (Teles et al., 2018). The link between flavonols and salt stress has been noted in the past, with increased levels of the flavonols quercetin and kaempferol being noted in *Apocynum venetum* seedlings following high salt exposure, as well as the upregulation of flavonol biosynthesis genes (*F3H*, *F3′H*, and *FLS*) coupled with the downregulation of general flavonoid biosynthesis genes (*CHS* and *CHI*) (Xu et al., 2020). This is consistent with observations by Walia et al. (2005), who noted that a rice genotype susceptible to salt stress had increased expression of *CHS*, *CHI*, *F3′H*, and *DFR* (the latter of which represents the branchpoint between flavonol and anthocyanin biosynthesis) in response to salinity stress compared to a tolerant line, correlating to the finding that susceptible varieties produced overall less flavonoids and phenolics when exposed to salt stress (Minh et al., 2016). Overexpression of *FLS* in *Arabidopsis* greatly increased the tolerance to salt stress, with the transgenic plants suffering far less membrane damage and displaying an overall improved phenotype under salt stress, with improved seed germination, growth, and chlorophyll content compared to the wild type (Guo et al., 2023). These findings seem to indicate that, although some flavonoids can increase tolerance to salinity stress, managing the competing branches of flavonoid biosynthesis is very important in inducing the desired phenotypic response.

Sulfated flavonoids have also been found to play a role in herbivore deterrence by reducing the attractiveness of sugars in *Zostera noltei* to the sea urchin *Paracentrotus lividus* (Casal-Porras et al., 2021), which is likely to be a secondary advantage conferred by these compounds in addition to their general function in aquatic higher plants. Sulfation may be important in modulating flavonoid function (Roubalová et al., 2015; Gidda and Varin, 2006) and has also been implicated in the signaling function of flavonoids —quercetin 3-sulfate reverts the auxin efflux inhibition caused by quercetin in *Flaveria bidentis*, a terrestrial dicot (Ananvoranich et al., 1994), though research regarding this function is limited (Chan et al., 2019), especially in marine angiosperms.

Overall flavonoid content has been observed to change according to the environmental conditions faced by seagrasses, changing with depth, location in the meadow, and season, with higher flavonoid contents generally being associated with increased environmental stress and competition (Casal-Porras et al., 2023; Sævdal Dybsland et al., 2021; Grignon-Dubois and Rezzonico, 2018). The relationship and ratios between flavonoids and non-flavonoid phenolics, especially rosmarinic acid, are typically assessed in these studies, with an increased flavonoid/rosmarinic acid ratio in *Z. marina* being attributed to more variable and stressful environments, such as the outskirts of a seagrass meadow when compared to the more sheltered interior (Sævdal Dybsland et al., 2021). Although flavonoids fulfill photoprotective functions in land plants (Agati et al., 2013), the importance of flavonoids in photoprotection differs between species of seagrasses, with some species seeming to rely more on rosmarinic acid for screening harmful radiation: in *Z. noltei*, there is a significant relationship between UV exposure and rosmarinic acid accumulation, but not flavonoid accumulation (Casal-Porras et al., 2023). Similarly, for *Halophila johnsonii*, flavonoid content was actually found to be higher in shaded plants, even though flavonoids were localized in epidermal cells where they could function as screening compounds (Gavin and Durako, 2012). The decrease in flavonoid content with increasing depth in *Z. marina* observed by Sævdal Dybsland et al. (2021) was therefore attributed to reduced general stress exposure with the buffering effect of the water, rather than reduced light exposure.

Spatial variation in flavonoid profiles can actually be used to ascribe certain species of seagrasses to distinct chemical phenotypes (Grignon-Dubois and Rezzonico, 2018), reflecting the importance of flavonoids in the ability of seagrasses to become widely distributed and combat different stresses along that distribution. In *Z. noltei*, chemotypes vary based on the dominant flavonoid, with 71%–83% of the flavonoid content in the Cadiz Bay population consisting of apigenin 7-sulfate, in contrast to the Arcachon Bay population where diosmetin 7-sulfate constituted 85%–93% of the total flavonoid content (Grignon-Dubois and Rezzonico, 2012). Both populations grow in intertidal meadows but are separated by approximately 1,000 km. The authors hypothesize that this dramatic difference is due to low expression of F3′H in the Cadiz population, which reflects the plasticity of flavonoid metabolism and the dramatic impact of a single gene on the metabolic flux of the pathway. The study was extended to include several other populations around the coast of Europe and North Africa (Grignon-Dubois and Rezzonico, 2018), which allowed them to define a third chemotype with high levels of apigenin 7-sulfate, diosmetin 7-sulfate, and luteolin 7-sulfate, though the ecological significance of these three chemotypes is not quite clear.

Changes in flavonoid and non-flavonoid phenolic content can also potentially act as an indicator of the health of seagrass meadows. As long as the phenolic profile is well understood under stable conditions, sudden deviations from the dynamics of certain indicator compounds can indicate shifts in the stress exposure of the meadow (Grignon-Dubois and Rezzonico, 2023; Astudillo-Pascual et al., 2021). As phenolic content can change quickly and specifically in response to certain stress conditions, such as the dynamics in the ratio of flavonoids to rosmarinic acid under different conditions in *Z. marina* studied by Sævdal Dybsland et al. (2021), the stresses faced by a certain population can be assessed based on these compounds and their relative abundances. Seagrasses require a degree of phenotypic plasticity to combat the multiple stresses they experience as mostly clonal populations (Pazzaglia et al., 2021), which makes polyphenolics and flavonoids very useful, as alternate branches of the pathway can be exploited to respond to differing stresses (Di Ferdinando et al., 2014). These ratios can also be used to easily asses the health of a seagrass meadow (Sævdal Dybsland et al., 2021), which can prove to be a very valuable tool in monitoring the level of stress experienced by a meadow due to anthropogenic climate change.

### 2.4 Flavonoids in response to climate change impacts on the marine environment

Anthropogenic climate change will affect marine ecosystems through ocean warming, acidification, marine heatwaves, and changes in storm patterns. Flavonoids, through their diverse stress response actions, can help mitigate the impact of some of these stress factors. Anthropogenic climate change is likely to increase seagrass exposure to toxic sulfides indirectly through enhancing the effects of eutrophication. Phytoplankton blooms not only limit the light availability for seagrass meadows and create hypoxic conditions but also increase organic matter mineralization in the sediment through enhanced sulfate reduction (Holmer and Bondgaard, 2001; Seidel et al., 2021). Ocean warming leads to further reduced oxygen levels (Schmidtko et al., 2017), further favoring anaerobic metabolic pathways and thereby increasing sulfite levels (Broman et al., 2017). Seagrass species that produce sulfated flavonoids may therefore be more resilient than other species in areas afflicted by eutrophication, leading to changes in distribution and meadow composition. Increased levels of sulfate stress resulting from eutrophication might have an impact on flavonoid signaling if this exists in seagrasses (Roubalová et al., 2015). Upregulation of sulfated flavonoid production could divert more flavonoid pathway intermediates which could interfere with other flavonoid functions, which may require more investment in flavonoid metabolism.

An interesting and troubling implication of climate change on seagrass survival is the reduction of phenolic compounds in response to ocean acidification (Arnold et al., 2012). As has been discussed thus far, seagrasses employ flavonoids and other phenolic compounds as versatile stress response compounds and increase flavonoid levels in more stressful, less stable environments. While increased atmospheric CO_2_ increases the phenolic content of terrestrial plants, seagrasses growing near volcanic CO_2_ vents experiencing low pH coupled with high CO_2_ levels display a loss of phenolic compounds, which makes them more vulnerable to herbivory (Arnold et al., 2012). This vulnerability to herbivory due to alterations in secondary metabolism is made more concerning in light of the shifting range of herbivores in response to climate change, with tropical herbivores shifting poleward and establishing in temperate seagrass meadows (Campbell et al., 2024). Herbivores can greatly affect the health of seagrass meadows and alter the food web by feeding directly on seagrasses instead of seagrass detritus (Lal et al., 2010; Longo, 2024; Hyndes et al., 2016; Campbell et al., 2024). The degree of this loss of phenols does seem to vary between populations, however, as found by a study comparing the metabolic response to elevated CO_2_ of two eelgrass populations (Zayas-Santiago et al., 2020). This study found that, while the primary metabolism of both populations benefited from increased CO_2_ concentrations, the population from a colder climate displayed less reduction in defensive compounds derived from the shikimate pathway, including phenolics. The spatial variation in phenolic content in seagrass species may therefore be an important factor in the perseverance of some seagrass meadows and populations over others in the acidifying and warming oceans.

The impact of the shift in herbivore distributions in response to ocean warming on seagrass species will also be affected by the accumulation of sulfated flavonoids. Sulfated flavonoids act as herbivore deterrents in some seagrass species (Casal-Porras et al., 2021), while other species lack these compounds (Grignon-Dubois and Rezzonico, 2018; Astudillo-Pascual et al., 2021), which could leave them vulnerable to herbivory. Seagrasses that do not produce sulfated flavonoids would therefore be confronted with a combination of increased sulfate toxicity and increased herbivory due to ocean warming, which would place them at a competitive disadvantage against other species. Additional monitoring would therefore be necessary for these species, which may have already been threatened due to anthropogenic activity. It is possible for new flavonoid sulfotransferase enzymes to arise within these species convergently, especially as ocean warming is expected to increase the rate of flowering and sexual reproduction in some seagrass species (Marín-Guirao et al., 2019; García-Escudero et al., 2024), which provides more opportunity for recombination from which improved genetic variants may arise. Alternatively, seagrass species which lack sulfated flavonoids may utilize alternative flavonoids or phenolpropanoids for herbivore deterrence and managing the damage caused by sulfates, which can lead to more distinct chemotypes. Shifts in flavonoid metabolism in species lacking sulfated flavonoids can be used to monitor how well different populations are responding to these stress factors and prioritize conservation efforts accordingly.

Seagrasses need to be able to adjust to fluctuations in salinity, especially those growing in estuarine environments where these fluctuations can be significant and rapid (Touchette, 2007). Climate change exacerbates these salinity changes beyond normal levels, with heavy rainfall and flooding leading to the loss of seagrass meadows due to increases in turbidity and decreases in salinity (Campbell and McKenzie, 2004; Chollett et al., 2007), while accelerated evaporations and influx of saltwater into lagoons from rising sea levels will likely lead to increased salinity (Jakimavičius et al., 2018; Shen et al., 2022). Flavonols may therefore be a flexible tool for seagrasses to tolerate the stress associated with increased salinity fluctuations caused by anthropogenic climate change. Flavonol metabolism is often in competition with anthocyanin metabolism, with the two pathways competing for pathway intermediates, metabolon interactions, and transcription factors (Owens et al., 2008; Luo et al., 2016; Yuan et al., 2016; Crosby et al., 2011). Anthocyanin production is likely to decrease under conditions of ocean warming, as this pathway is upregulated in response to cold stress (Sullivan and Koski, 2021), which would allow for increased levels of flavonols under these conditions. Increased levels of evaporation in estuarine habitats, which would lead to increased salt concentrations, would also increase the level of UV exposure faced by these seagrasses. Both flavonols and anthocyanins are antioxidants involved in combatting UV damage, but under conditions of ocean warming and increased salinity in estuaries, flavonols may become more important than anthocyanins in fulfilling a UV-protective function.

## 3 Phenylpropanoid and flavonoid biosynthesis genes in seagrasses

**Figure 2:**
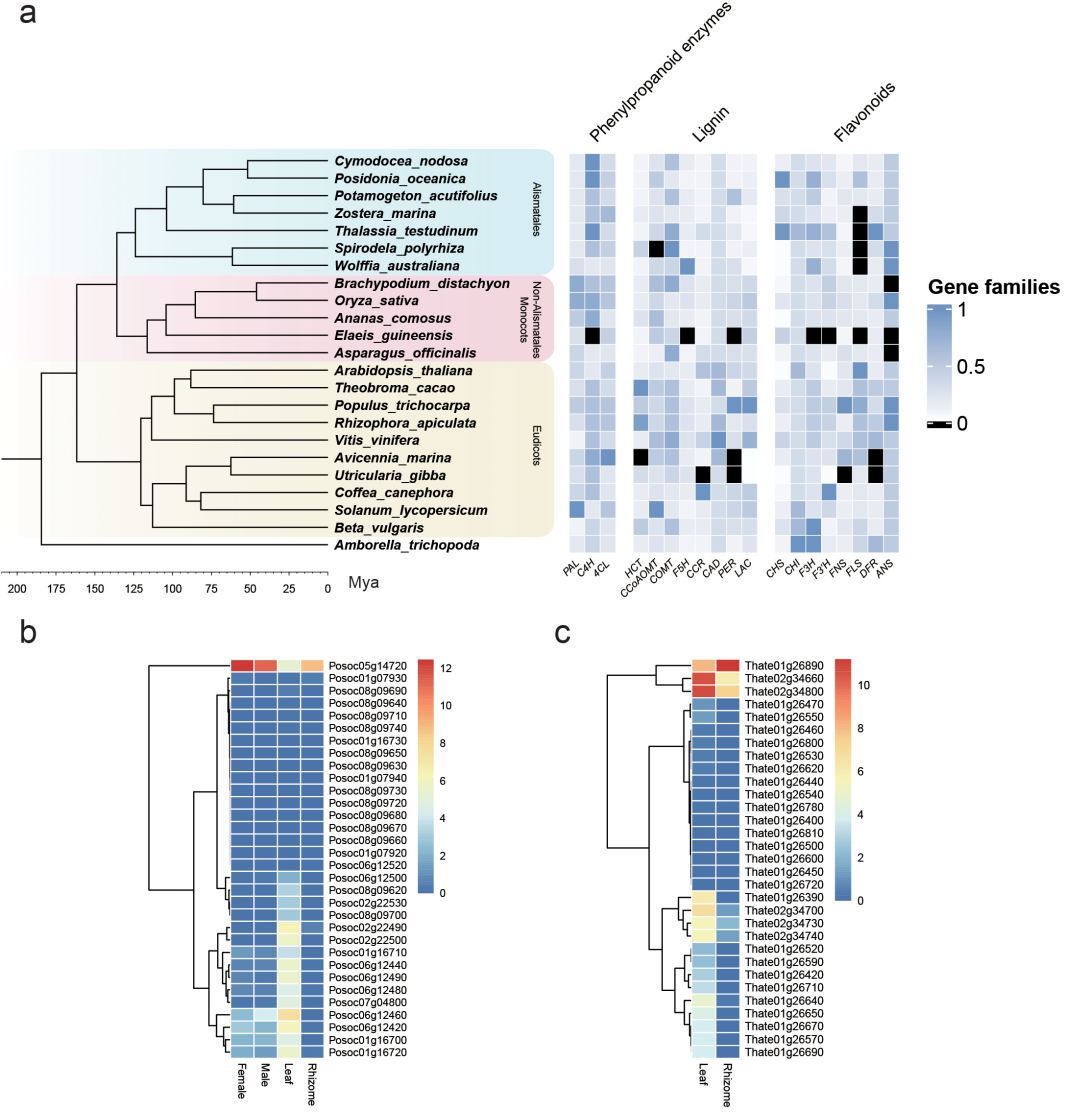
Phenylpropanoid and flavonoid biosynthesis genes in seagrasses. Transcriptome data from Ma et al. (2024) for the annotation of four seagrass genomes (**Supplementary Data Sheet 1**). **(A)** Changes in phenylpropanoid gene families in four seagrass species compared to various other terrestrial and aquatic lineages. Seagrasses in this figure are represented by *C. nodosa*, *P. oceanica*, *Z. marina*, and *T. testudinum*, with other members of the Alismatales being freshwater species. Gene family numbers are normalized by dividing the gene count number of each species by the highest copy number for that family. **(B)** Gene expression levels of CHS genes in various organs of *P. oceanica* and the expression values are scaled by log2(TPM + 1). **(C)** Gene expression levels of CHS genes in various organs of *T. testudinum* and the expression values are scaled by log2(TPM + 1).

**Figure 2** shows the relative number of gene family members represented in various seagrass and non-seagrass lineages for genes involved in phenylpropanoid metabolism, from a meta-analysis of the annotation data published by Ma et al. (2024). In terms of general phenylpropanoid genes, seagrasses generally possess genes in copy numbers similar to those of other aquatic and non-aquatic plants, showing that there is no significant change in this pathway in the shift to marine ecosystems. The only exception is the enzyme LAC, which is present in fewer copies in both the examined seagrass and freshwater-floating lineages compared to land angiosperms. This enzyme is involved in polymerizing monolignols to produce lignin (Dong and Lin, 2021), with lignin being attributed as another important trait in the evolution of land plants, providing mechanical support and allowing for the advent of vascular tissue (Lei, 2017). Aquatic plants have been found to have lower levels of lignin compared to land plants (Rabemanolontsoa and Saka, 2013), which relates to their increased flexibility (Asaeda et al., 2005). This allows these plants to move with the substrate and suffer reduced mechanical damage from currents and tides, making it an important adaptation to submerged life retained by seagrasses (Ma et al., 2023).

As can be seen in **Figure 2**, *P. oceanica* and *T. testudinum* have an unusually high number of CHS gene representatives. The copy number of this gene varies between species, with *Arabidopsis thaliana* possessing a single copy, *Ipomoea purpurea* three (Durbin et al., 1995), and *Glycine max* eight (Tuteja et al., 2004). The different members of the CHS gene family for a species are often subject to differential spatial–temporal regulation (Deng et al., 2014), allowing for fine control over flavonoid production. Duplication and differentiation of the CHS gene allows for neofunctionalization and subfunctionalization, which Durbin et al. (2000) suggest to be an important strategy in plant adaptation and evolution. Stilbene synthases, for example, have evolved from CHS several times independently, as only a few missense mutations are required to confer stilbene synthase activity to CHS (Tropf et al., 1994). CHS can also produce other polyketide products which cannot be used for further flavonoid biosynthesis —this catalytic promiscuity is likely what allows for the advent of stilbene synthases and other plant-specific polyketide synthases from CHS (Weng and Noel, 2012). The high copy number of CHS in these two seagrass species can therefore represent the need for very fine control over flavonoid production under different environmental conditions or could represent functional diversification of this gene family in these lineages. In *Z. marina*, 11 CHS homologs were identified in a 2021 paper (Ma et al., 2021a), which could be divided into three clades. These homologs were all found to be functional in producing naringenin chalcone with differing tissue-specific expression patterns and light responses. Studies focusing on chalcone synthases in seagrasses are limited beyond this one and would definitely prove interesting in light of the unusually high copy number found in *P. oceanica* and *T. testudinum.* Based on expression data collected for the annotation of the *P. oceanica* and *T. testudinum* genomes (Ma et al., 2024), the different CHS copies do display tissue-specific expression differences, as shown in **Figures 2B, C**, but more data are necessary to capture the full expression profiles of these genes under different conditions.

It is possible that seagrass species with a wider set of CHS copies could respond better to stressful conditions imposed by climate change, due to more specific control over flavonoid upregulation. As discussed earlier, ocean acidification can lead to reduced phenolic content as primary metabolism is favored (Arnold et al., 2012). This is less of a concern for plants which are able to quickly upregulate flavonoid biosynthesis in the appropriate tissue type when exposed to certain stress conditions, such as increased sulfides or sudden increases in temperature.

Another interesting feature is the absence of *FLS* orthologs in *Z. marina* and *T. testudinum*, especially regarding the prevalence of sulfated flavonoids in seagrasses. Flavonol synthase is required for the production of flavonols, which along with flavones are generally the favored substrates for sulfotransferases (Teles et al., 2018). The lack of this gene could therefore point to lineage-specific differences in sulfated flavonoid composition relating to flavone and flavonol levels. As discussed previously, flavonols are associated with salt stress, as well as functioning in various stress responses, inhibiting auxin transport and modulating plant growth under stress conditions (Daryanavard et al., 2023). Flavonols are also very effective as antioxidants and UV-B screening compounds (Pollastri and Tattini, 2011), which makes them useful for plants occupying stressful and variable habitats and potentially very important for combatting the stresses imposed by climate change (Laoué et al., 2022). The absence of *FLS* orthologs could therefore have interesting implications for the function of flavonols in these lineages and their ability to deal with changing environmental conditions, which warrants further investigation.

The high number of DFR orthologs in *T. testudinum* has interesting implications for anthocyanin biosynthesis in this lineage compared to the other examined seagrasses. DFR occupies the branchpoint toward anthocyanin biosynthesis, in opposition to FLS directing dihydroflavonols toward flavonol biosynthesis, as can be seen in **Figure 1**. Different DFR copies are associated with spatial–temporal control of anthocyanin accumulation (Li et al., 2017), with anthocyanin accumulation often being a response to high light and low-temperature cues (Ahmed et al., 2015; Christie et al., 1994; Ma et al., 2021b; Jiang et al., 2016). The high copy number of DFR and the loss of FLS might therefore imply that anthocyanins play a more important role in these stress responses in *T. testudinum* compared to other seagrass lineages.

## 4 Anthocyanins

**Figure 3:**
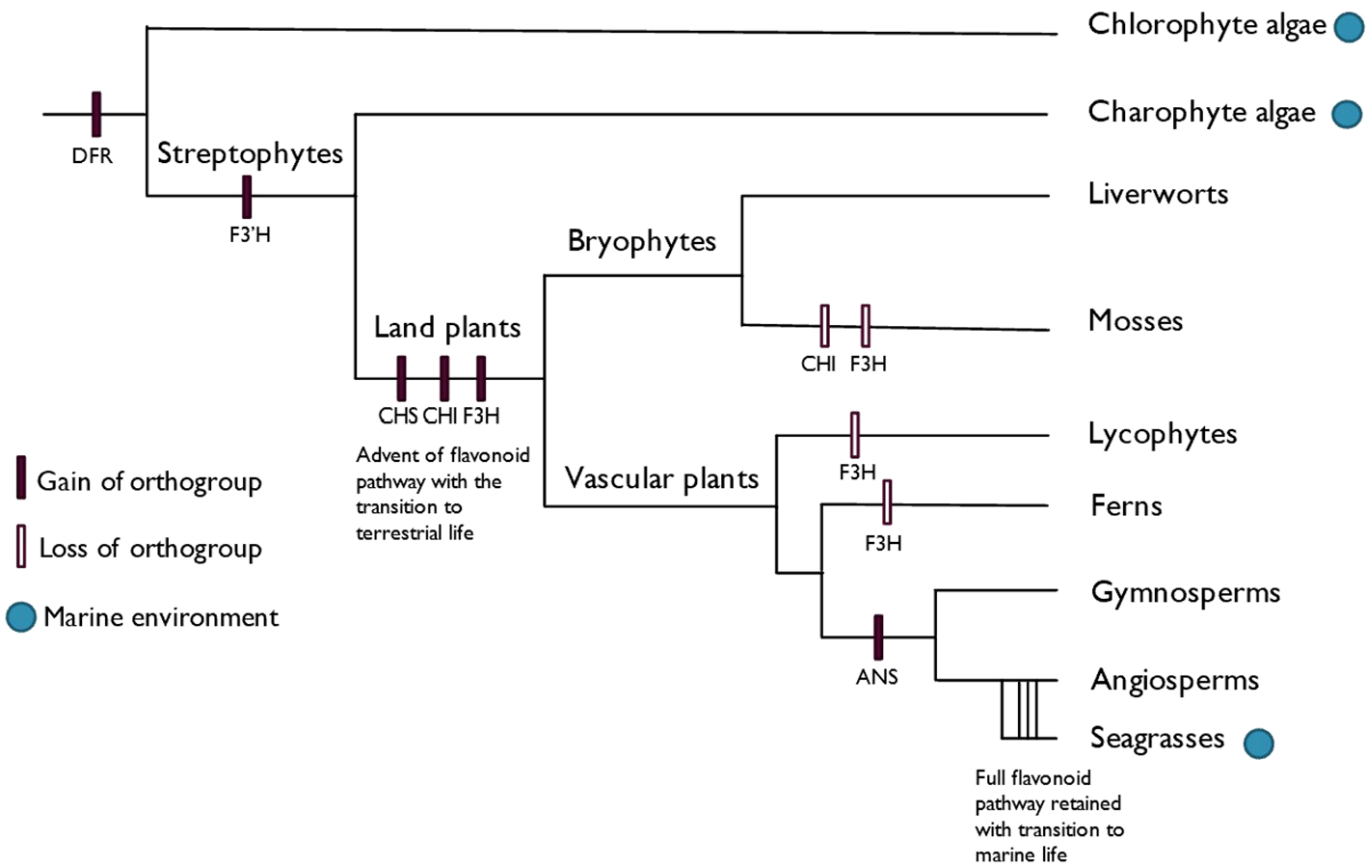
Evolution of the anthocyanin biosynthesis pathway, adapted from Piatkowski et al. (2020). The origin of each orthogroup of the anthocyanin biosynthesis genes is shown, with the complete pathway being limited to the seed plants with the advent of ANS. Seagrasses therefore have a host of genes at their disposal which other photosynthetic marine organisms lack.

Anthocyanins, as a subclass of flavonoids, may be particularly interesting from a functional point of view in seagrasses, due to them only arising within seed plants (Piatkowski et al., 2020), which makes their function within marine ecosystems interesting. Anthocyanin biosynthesis and its underlying genes have been well characterized for a few decades, with the visual detection of anthocyanin presence and absence allowing for easy detection of mutations in the biosynthetic and regulatory genes. The full anthocyanin pathway is limited to seed plants, with ANS only arising in their common ancestor (Piatkowski et al., 2020), as shown in **Figure 3**. Seagrasses have retained the ability to synthesize anthocyanins and benefit from them as versatile antioxidants and UV-screening compounds, which non-angiosperm marine organisms do not possess. Like other flavonoids, anthocyanins function in various ROS-producing stress responses, which most likely predates their function in pollinator signaling (Rudall, 2020).

Older lineages, such as the bryophytes, produce other red- and purple-pigmented flavonoids, which perform similar protective functions to anthocyanins (Aigner et al., 2013; Ben Saad et al., 2017). Some of these pigments were once thought to be anthocyanidins (Kunz et al., 1993), leading to the hypothesis that anthocyanins arose with the shift of plants to terrestrial habitats along with the first flavonoids, but these pigments have since been found to be a chemically distinct class of flavonoids called auronidins (Berland et al., 2019). This might be a case of convergent evolution, with the compounds having antioxidant activity (Ben Saad et al., 2017) and being produced in response to similar environmental stresses (Snell et al., 2009).

Although the mechanism of anthocyanins in combating oxidative stress is not well understood, the correlation between anthocyanin production and reduced susceptibility to oxidative stress has been observed many times: reduced expression of anthocyanin genes results in increased hydrogen peroxide accumulation after exposure to cold stress (Wang et al., 2013); anthocyanin-deficient mutants show increased chlorophyll and membrane damage and decreased antioxidant capability when exposed to photooxidative stress (Shao et al., 2008); and there is upregulation of anthocyanin biosynthetic genes and their corresponding regulatory genes in response to ROS accumulation (Xu et al., 2017). There is a host of evidence for the importance of anthocyanins in cold stress, with genes specific to anthocyanin production being upregulated under cold stress conditions (He et al., 2020), accumulation of anthocyanin pigments under these conditions (Ahmed et al., 2015; Christie et al., 1994), anthocyanin-deficient mutants being more susceptible to cold stress, and anthocyanin-free tissues accumulating more reactive oxygen species (Meng et al., 2015) and suffering more damage to photosynthetic efficiency than purple-pigmented tissues (Zhang et al., 2019). Reduced expression of anthocyanin biosynthetic genes through RNAi in purple sweet potato (*Ipomea batatas*) resulted in slower recovery after exposure to cold stress, with increased membrane damage and accumulation of ROS (Wang et al., 2013).

Photoprotection is another important function of anthocyanins, acting both as a sunscreen by absorbing certain wavelengths of light and scavenging free radicals produced by UV radiation (Zeng et al., 2010; Merzlyak et al., 2008; Hughes et al., 2005). Photoinhibition is the result of damage to the reaction center of photosystem II (PSII) caused by strong light, with photosynthetic efficiency suffering when the rate of damage exceeds the rate of *de-novo* PSII protein synthesis (Adir et al., 2003; Murata et al., 2007). Anthocyanins can be produced in response to high light or constitutively produced in certain vulnerable tissues, such as young leaves, to mitigate the impact of high light on photosynthetic efficiency (Ma et al., 2021b; Karageorgou and Manetas, 2006; Zhang et al., 2016).

Anthocyanin biosynthesis is capable of responding to a variety of stress conditions, with multiple hormonal signals being able to influence anthocyanin production (Lafountain and Yuan, 2021). The late genes of the biosynthetic pathway (DFR, ANS, and 3UGT), which are specific to anthocyanin biosynthesis, are all regulated by an MBW transcriptional activation complex (Quatrocchio et al., 1993), which consists of three proteins. The first is an R2R3 MYB protein, first identified as the maize Myb domain C1 protein that recognizes two consensus sequences in the promoters of the late biosynthesis genes (Sainz and Chandler, 1997). A basic helix-loop-helix (bHLH) transcription factor also plays a role in promoter recognition with a distinct consensus sequence (Ludwig et al., 1990). Plants possess multiple copies of these genes which show tissue-specific and stress-responsive expression (Xu et al., 2014): regulation of the late biosynthesis genes is therefore achieved through the regulation of these proteins. Both the MYB and bHLH families of transcription factors have undergone dramatic expansion in higher plants, and their increase in diversity is associated with an increase in metabolic complexity (Feller et al., 2011). The third protein is a WD40 repeat protein that plays no role in promoter recognition.

Hormonal control of anthocyanin production often relies on the sequestration of the R2R3 MYB and bHLH members of the ternary complex by proteins such as JAZ and DELLA (Qi et al., 2011, Qi et al., 2014). Hormones, such as jasmonate and gibberellic acid, can activate anthocyanin biosynthesis through triggering the ubiquitination of JAZ and DELLA, respectively, resulting in the degradation of these repressor proteins by the 26S proteasome and the formation of the MBW complex. The recent review article by Lafountain and Yuan (2021) gives a good overview of the current knowledge of the negative regulation of anthocyanin biosynthesis, describing over a dozen mechanisms. At the end of the review, they question why so many negative regulators of anthocyanin biosynthesis have evolved: anthocyanins have a range of functions and are produced in response to an array of stimuli, so why evolve repressors instead of activators? The authors point out that many of these repressors function in a similar manner, being degraded in response to a signal to allow for the activation of the pathway genes. This double-negative logic (Davidson and Levine, 2008) removes the need for the constituent proteins of the MBW complex to obtain novel promoter elements in order to respond to a certain stimulus, and the degradation of a repressor allows for rapid upregulation of anthocyanin gene expression upon signal reception. This allows for fine control over anthocyanin biosynthesis under stress conditions and highlights the flexibility of flavonoid metabolism.

### 4.1 Roles of anthocyanin in abiotic stress responses of seagrasses

While marine environments can be more stable than terrestrial ones with water screening out UV-B radiation and modulating the temperature, intertidal areas can be particularly variable, with strong fluctuations in light intensity requiring photosynthetic organisms to maximize photosynthetic efficiency under low light conditions and protect photosynthetic machinery under extreme levels of irradiance (Kohlmeier et al., 2017; Léger-Daigle et al., 2022). Anthocyanin biosynthesis is subject to extensive negative regulation, as reviewed by Lafountain and Yuan (2021), often involving various plant hormones, which allows for rapid increase in anthocyanin production upon the reception of a signal. This, combined with their UV-screening ability, makes them very useful for seagrasses which can experience variable levels of UV exposure, on account of differences in depth (Dattolo et al., 2014), meadow density (Schubert et al., 2015), and tides (Kaewsrikhaw and Prathep, 2014). Leaf reddening has been observed in a limited number of seagrass species growing in intertidal regions and shallow waters and is associated with stress exposure (Novak and Short, 2010). Anthocyanin production can be upregulated for photoprotection when exposed to high light conditions (Ma et al., 2021b), while energy is not wasted in conditions where UV-screening mechanisms are unnecessary.

The accumulation of anthocyanins to compensate for the shortcomings of other photoprotective mechanisms has also been observed in terrestrial plants (Zhu et al., 2018). In several species of seagrasses, leaves develop red coloration due to anthocyanin accumulation following high light exposure, with the anthocyanins acting as UV-B- screening compounds or compensating for reduced capacity of photoprotective strategies (Buapet et al., 2017; Zhu et al., 2018). The depth and density of the seagrass meadow also influences the degree of light exposure and thereby the degree of anthocyanin accumulation (Kaewsrikhaw et al., 2016; Dattolo et al., 2014). This variation in pigment content between seagrasses can actually discriminate between species by remote sensing and can therefore be a useful tool in monitoring the health of seagrass meadows (Fyfe, 2003). In *T. testudinum*, foliar anthocyanin accumulation can be a permanent trait or induced in response to light (Novak and Short, 2012), with red leaves displaying increased levels of UV-absorbing compounds besides anthocyanins and maintaining better photosynthetic efficiency than green leaves at midday (Novak and Short, 2011). Interestingly, a study by Kaewsrikhaw and Prathep (2014) found that anthocyanin content in *Halophila ovalis* was significantly higher during the rainy season, when light intensity was much lower. This contradicts the hypothesis of the involvement of anthocyanins in UV screening in seagrasses, which seems to hold for some other species, like *Zostera capricorni* and *T. testudinum* (Fyfe, 2003; Novak and Short, 2011). While anthocyanins are useful in photoprotection in seagrasses, green leaves do not necessarily suffer greater photoinhibition than red leaves in all seagrass species, as other photoprotective mechanisms can still be effective in high light environments (Buapet et al., 2017; Dattolo et al., 2014). A later study by the same authors (Kaewsrikhaw et al., 2016) found that *H. ovalis* growing in intertidal areas had higher anthocyanin content compared to individuals growing in subtidal areas. The latter population also showed increased chlorophyll content to mitigate the effects of lower light at greater depths. This observation once again supports the role of anthocyanins in photoprotection and UV screening and somewhat contradicts their earlier thoughts regarding anthocyanin accumulation during the rainy season (Kaewsrikhaw and Prathep, 2014). This might in fact be important for managing cold stress during the rainy season, which is a known function of anthocyanins in some plants (Ahmed et al., 2015; Christie et al., 1994; Schulz et al., 2016).

In the shift to aquatic environments, some seagrasses like *Z. marina* have lost genes involved in UV sensing and resistance, due to the screening effect of seawater rendering these genes redundant (Olsen et al., 2016). Among these lost genes are photoreceptors, specifically CRY2, which play an important role in upregulating anthocyanin biosynthesis genes and anthocyanin accumulation under high light conditions (Jiang et al., 2016), which results in some species of seagrasses being especially vulnerable to photoinactivation of the oxygen-evolving complex (OEC) under harsh light conditions (Wang et al., 2022; Zhao et al., 2021).

Anthocyanins provide a mechanism of resilience to cold stress and photooxidative damage, but as ocean water provides a buffering effect on both of these stresses, seagrasses may have a smaller set of stress response mechanisms (Olsen et al., 2016; Zhang et al., 2022). In a study by Zhang et al., 2022 it was found that low temperatures combined with high light damaged the photosynthetic machinery of three tropical seagrass species (*Enhalus acoroides*, *Thalassia hemperichii*, and *Cymodocea rotundata*), limiting their distribution to the Indo-Pacific convergence regions. *Enhalus acoroides* and *T. hemperichii* do experience leaf reddening (Apichanangkool and Prathep, 2014), and anthocyanins are probably an important mechanism in mitigating cold stress during emersion combined with high light in the absence of other mechanisms. As anthocyanins are cold-induced, they have been found to decrease under temperature increases in terrestrial plants (Sullivan and Koski, 2021), though most research pertaining to the subject is focused on anthocyanin content of grapes for the wine industry. Ocean warming and marine heatwaves may therefore have adverse effects on anthocyanin accumulation in seagrasses, which may be especially detrimental to species that rely heavily on anthocyanins as a photoprotective and cold stress resistance mechanism.

### 4.2 Anthocyanins in seagrass reproduction

Seagrasses generally reproduce asexually through stolons, though sexual reproduction does occasionally occur, which is important in maintaining standing genetic variation in populations; however, the success rate is poor. Seagrasses generally rely on hydrophilous pollination and engage in various strategies to avoid self-pollination, including dioecy, reduced perianths, and separation of male and floral structures in monoecious species (Van Tussenbroek et al., 2016). Ocean warming and marine heatwaves affect the flowering behavior of different seagrass species differently, with the flowering of cold-adapted *P. oceanica* seemingly being triggered by marine heat waves (Marín-Guirao et al., 2019; García-Escudero et al., 2024), while *Z. marina* has displayed a decrease in flowering frequency and intensity as oceans warmed (Qin et al., 2020). Although the flowers of seagrasses are generally green or pale yellow, in accordance with the abiotic pollination strategy, a transcriptomics study in *P. oceanica* actually found anthocyanin biosynthesis genes to be upregulated in floral tissues, which results in a slight red pigmentation of the male reproductive structures appearing prior to pollen release (Entrambasaguas et al., 2017). The purpose of this anthocyanin accumulation is currently unclear, but anthocyanin production is generally downregulated in response to increased temperatures (Sullivan and Koski, 2021), which will likely lead to a loss of red pigmentation in these flowers as oceans continue to warm.

Anthocyanins are generally produced in the flowers of angiosperms to act as an attractive cue for pollinators, allowing them to be distinguished from the surrounding foliage visually and thermally (Harrap et al., 2017; Takács et al., 2009; Seymour and Matthews, 2006). The discovery of invertebrate-mediated pollination in *T. testudinum* (Van Tussenbroek et al., 2012, Van Tussenbroek et al., 2016), in combination with the discovery of floral anthocyanin accumulation in *P. oceanica*, indicates that the possibility of anthocyanins functioning in pollinator attraction in seagrasses cannot be ruled out. Alternatively, the floral anthocyanin accumulation may act as a strategy to deter herbivores, as anthocyanin accumulation can signal metabolic investment in a tissue or decrease its palatability (Gould, 2004). Though biotic pollination was until recently believed to not occur in aquatic ecosystems at all, this discovery in this one species of seagrass spurred additional research regarding pollination benefits arising from other observed biotic interactions involving red algae (Lavaut et al., 2022), which indicates that invertebrate pollination in aquatic ecosystems may be very ancient. Ocean warming is predicted to shift the ranges of herbivores poleward, which may negatively impact seagrass meadows losing invertebrate pollinators and positively affect the sexual reproduction of seagrass meadows that are introduced to this new layer of interaction. The accompanying loss of anthocyanins in the reproductive tissues of some seagrass species may make them more difficult to seek out by potential pollinators or make them more palatable to herbivores, with both scenarios negatively impacting sexual reproduction.

## 5 Priorities for future research

The availability of genomic data from representatives of the four seagrass families (the *Posidoniaceae, Zosteraceae, Hydrocharitaceae,* and *Cymodoceaceae*) provides a valuable framework for future research into the role of flavonoids and anthocyanins in these marine organisms. A priority would be to establish a genomics database for seagrasses, where gene family classifications and protein functions can be updated in real time with the sequencing of additional seagrass species. Gene catalogs from RNA sequencing projects can be especially useful in this regard. Genomic technologies also provide a powerful framework for studying the diversity of individual seagrass species at the population level by linking chemotyping with gene expression profiling and proteomics. Documenting these molecular differences between seagrass populations can also help in understanding and predicting differing responses to stress imposed by climate change.

This review highlights some areas for immediate inquiry. The expansion of the *CHS* gene family in *T. testudinum* and *P. oceanica* compared to other seagrass species can be investigated by determining whether this expansion is present in other species of these families and evaluating neo- or subfunctionalization of flavonoids by metabolite and RNA profiling of different tissues and populations of each species. It would also be interesting to examine whether species with large CHS gene families are able to use flavonoids more effectively in dealing with stress. The absence of *FLS* orthologs in *Z. marina* and *T. testudinum* points to an absence of flavonols in these species. These compounds have been implicated in the detoxification of sulfur in seawater and play important roles in the management of salt stress in many species. Metabolite profiling would confirm the absence of these compounds, which could have interesting implications for how these species will respond to changes in salt concentration caused by more frequent storms. A further area of investigation is the high number of DFR orthologs in *T. testudinum*, which implies an important role of anthocyanins in this species, as DFR represents the first committed step in anthocyanin biosynthesis. Ocean warming will probably lead to a decrease in anthocyanin content, which will likely affect species that rely on anthocyanins more heavily more so than species that employ additional stress response mechanisms. Experiments testing the effects of UV radiation under warmer conditions on seagrasses that undergo leaf reddening will help to elucidate the potential impact of ocean warming on photoprotection efficiency. The vulnerability of seagrass reproductive structures to herbivory under warming conditions could also help to understand the function of anthocyanin accumulation in these organs.

## 6 Conclusions

There are several take-home messages from this review on flavonoids in seagrasses and the impacts of climate change.

The ability to produce flavonoids may have aided seagrasses in adapting to aquatic environments, even though these compounds were important for the shift to terrestrial ecosystems in the ancestors of land plants.

- Flavonoids are useful compounds in variable habitats, due to the interplay between different pathways allowing for reactions to different types of stress.
- There is an interplay between flavonoids and other simple phenolic compounds in seagrasses to manage different types of stress, with flavonoid content generally being higher in more variable or stressful environments.
- Seagrasses that produce sulfated flavonoids may be more resilient to increased eutrophication resulting from anthropogenic climate change.
- The role of flavonols in seagrasses is not well understood but may have important implications for dealing with changes in salinity.
- Ocean acidification may impact secondary metabolism, including flavonoid production, negatively.
- Flavonoid content can be used as a measure for assessing the health of seagrass meadows, with changes in flavonoid content reflecting the degree of stress faced by the plants, which may prove to be especially important in understanding how different populations are affected by climate change.
- Seagrasses display several interesting genetic features relating to flavonoid metabolism, with a very high number of CHS orthologs in *T. testudinum* and *P. oceanica* and the absence of FLS orthologs in the former and *Z. marina*, which warrants further research.
- Anthocyanin accumulation may allow for flexible protection against UV radiation in seagrasses, with the loss of other protective screening mechanisms. However, the precise function of anthocyanin accumulation in certain species of seagrasses remains unclear.
- Ocean warming will likely have a more severe impact on seagrass species that heavily rely on anthocyanins as a method of photoprotection.
- Understudied marine biotic interactions may have important implications for flavonoid and anthocyanin accumulation and chemistry in seagrasses, and ocean warming will likely change the patterns of these interactions.
