# Supplementary material for: Flavonoids and anthocyanins in seagrasses: implications for climate change adaptation and resilience
Source: Front Plant Sci. 2025 Jan 28;15:1520474. doi: 10.3389/fpls.2024.1520474 (PMC11810914; doi:10.3389/fpls.2024.1520474)
Supplement: Supplementary file 1 [file DataSheet1.docx]

All data used in this review was originally made available in the article “Seagrass genomes reveal ancient polyploidy and adaptations to the marine environment” by Ma et al., published in Nature Plants in 2024.

All assemblies and annotations for all seagrass species discussed in the current paper can be found at https://bioinformatics.psb.ugent.be/gdb/seagrasses/. The transcriptome data (including raw data and clean data) and sequencing QC reports for *C. nodosa* can be found at https://genome.jgi.doe.gov/portal/pages/dynamicOrganismDownload.jsf?organism=Cymnodnscriptome_2, the transcriptome data and sequencing QC reports for *P. oceanica* can be found at https://genome.jgi.doe.gov/portal/pages/dynamicOrganismDownload.jsf?organism=Posocenscriptome_2, the transcriptome data and sequencing QC reports for *T. testudinum* can be found at https://genome.jgi.doe.gov/portal/pages/dynamicOrganismDownload.jsf?organism=Thatesnscriptome_4 and the transcriptome data for *Z. marina* are from ref. 15. For the public databases, the RFAM database v.14.7 can be downloaded at https://ftp.ebi.ac.uk/pub/databases/Rfam/14.7/, the UniProt database can be accessed from the web at http://www.uniprot.org and downloaded from http://www.uniprot.org/downloads and the NCBI nucleotide database can be accessed via https://www.ncbi.nlm.nih.gov/.
